# Examining the Roles of Reasoning and Working Memory in Predicting Casual Game Performance across Extended Gameplay

**DOI:** 10.3389/fpsyg.2017.00203

**Published:** 2017-03-07

**Authors:** Michael B. Kranz, Pauline L. Baniqued, Michelle W. Voss, Hyunkyu Lee, Arthur F. Kramer

**Affiliations:** ^1^Department of Psychology, Beckman Institute for Advanced Science and Technology, University of Illinois at Urbana ChampaignUrbana, IL, USA; ^2^Helen Wills Neuroscience Institute, University of California BerkeleyBerkeley, CA, USA; ^3^Department of Psychological and Brain Sciences, University of IowaIowa City, IA, USA; ^4^Posit Science, Brain Plasticity InstituteSan Francisco, CA, USA

**Keywords:** casual games, working memory, reasoning, fluid intelligence, skill acquisition

## Abstract

The variety and availability of casual video games presents an exciting opportunity for applications such as cognitive training. Casual games have been associated with fluid abilities such as working memory (WM) and reasoning, but the importance of these cognitive constructs in predicting performance may change across extended gameplay and vary with game structure. The current investigation examined the relationship between cognitive abilities and casual game performance over time by analyzing first and final session performance over 4–5 weeks of game play. We focused on two groups of subjects who played different types of casual games previously shown to relate to WM and reasoning when played for a single session: (1) puzzle-based games played adaptively across sessions and (2) speeded switching games played non-adaptively across sessions. Reasoning uniquely predicted first session casual game scores for both groups and accounted for much of the relationship with WM. Furthermore, over time, WM became uniquely important for predicting casual game performance for the puzzle-based adaptive games but not for the speeded switching non-adaptive games. These results extend the burgeoning literature on cognitive abilities involved in video games by showing differential relationships of fluid abilities across different game types and extended play. More broadly, the current study illustrates the usefulness of using multiple cognitive measures in predicting performance, and provides potential directions for game-based cognitive training research.

## Introduction

Video game websites (e.g., miniclip.com, addictinggames.com) offer hundreds of games across a variety of genres. These freely available, highly accessible, easy-to-learn games—often called *casual games*—provide a leisurely yet cognitively engaging activity even for people with limited video game experience. Whether maneuvering around obstacles to reach a door or exit, quickly collecting coins, or shooting down enemy ships, casual games challenge players' cognitive abilities in a variety of ways. Can we harness this potential for cognitive applications such as cognitive training? Addressing this question requires a deeper understanding of the cognitive processes involved in casual games over extended gameplay.

In a recent study, several of these freely available, web-based casual games were quantitatively evaluated in terms of their relationship with cognitive abilities (Baniqued et al., 2013). Specifically, participants completed a battery of cognitive tests and played several casual games for one short period of time (i.e., 20 min per game) while instructed to achieve the highest score or level. Performance on several games correlated with tests of *working memory* (WM), which relates to actively maintaining and manipulating information in mind (Baddeley, 1992), and *reasoning*, which relates to solving novel problems (also called fluid intelligence; Cattell, 1987). Although informative, this evaluation did not assess the relationships over a longer period of time such as extended gameplay over several sessions, which is common in both everyday use (http://www.casualgamesassociation.org) and in cognitive training research (Basak et al., 2008; Boot et al., 2010; Owen et al., 2010; Colom et al., 2012; Baniqued et al., 2014; Lee et al., 2015). Given that the relationship between cognitive abilities and games (or training tasks) motivates cognitive training research design (Jaeggi et al., 2010; Baniqued et al., 2013), evaluating how these relationships change after extended play for different types of games is important.

In one recent framework of complex skill acquisition, it is thought that individuals first form strategies in an effortful and error-prone process, and that performance is largely associated with fluid abilities (Fleishman, 1972; Woltz, 1987; Ackerman, 1988, 2005) such as working memory and reasoning. After initial learning, the relationship with fluid abilities becomes dependent on task consistency (Ackerman, 1988, 2005). In consistent task environments, individuals then tune and automatize strategies over time, leading to more efficient task performance; the association between task performance and fluid abilities decreases, while the association between task performance and the speed of strategy deployment (i.e., processing speed) increases. In contrast, in inconsistent or variable task environments, individuals must update strategies in response to changing task components, and task performance remains associated with fluid abilities over time. Thus, if the goal of a training program is to improve fluid abilities, inconsistent or variable cognitive training environments may be desirable or more optimal.

This framework has been commonly applied to understand complex skill acquisition on a range of complex tasks from short-term learning (Zhang et al., 2007), computer programming (Shute and Kyllonen, 1990), to commercial brain training games (Quiroga et al., 2009, 2011, 2015; Ackerman et al., 2010). However, applying the framework to casual games is more complex because many casual games are adaptive, where difficulty increases as the game progresses. For some games, difficulty increases with more complex obstacles and unique relationships to learn on each new level. For these particular games, players often start on the highest level reached from the previous sessions. This game structure requires players to learn novel rules and skills across multiple sessions of gameplay (*adaptive* across sessions). In contrast, other casual games involve repeating the same levels, with each session of gameplay starting at the same difficulty level (*non-adaptive* across sessions), but with each attempt involving increases in difficulty until performance limits are reached (e.g., a “game over”). In this study, we examine whether performance in these different types of casual games is differentially predicted by fluid abilities.

To measure fluid abilities, training studies have primarily used reasoning tasks (e.g., Ackerman, 1988) and working memory (WM) tasks (e.g., Woltz, 1987; Kyllonen and Stephens, 1990; Shute and Kyllonen, 1990). Starting with Kyllonen and Christal (1990), research has consistently identified a strong association between WM and reasoning (e.g., Engle et al., 1999; Colom et al., 2003; Conway et al., 2003; Ackerman et al., 2005). In one meta-analysis, WM and reasoning shared ~50% of their variance across studies (Kane et al., 2005). Due in part to this robust relationship, many cognitive training paradigms seek to improve fluid abilities, as measured with reasoning tasks, using tasks that tap working memory ability, with WM thought to reflect a more basic and fundamental process underlying reasoning ability (see Colom et al., 2010; Jaeggi et al., 2010).

Despite their strong relationship, the two constructs are not considered isomorphic (Ackerman et al., 2005; Kane et al., 2005). Indeed, several areas of research illustrate the importance of the non-overlapping variance of WM and reasoning. In one domain—academics—higher reasoning and WM abilities independently predicted academic achievement test scores in children (Alloway and Alloway, 2010; Dumontheil and Klingberg, 2012). In the laboratory, WM and reasoning uniquely predicted performance on problem solving tasks placing high demand on WM (3–8 disk Tower of Hanoi problems), but only reasoning predicted performance on problem solving tasks lower in WM demand (2–5 move Tower of London problems; Zook et al., 2004). Moreover, increasing the demand on WM tasks seemed to have no effect on the relationship between reasoning and working memory (Unsworth and Engle, 2005; Salthouse et al., 2008).

The current study assesses the relationship between abilities implicated in skill acquisition and casual game performance across extensive gameplay^1^. This inquiry will shed light on the cognitive components of casual games and help us understand how a leisure time activity pursued by an increasing number of individuals is associated with aspects of cognition. A better understanding of these associations may ultimately lead to more informed use of casual games for cognitive training research and more generally, better informed applications of computer-based games for other interventions or real-world situations.

To this end, we leveraged data from two groups of participants who played casual games over multiple sessions as part of a cognitive training study (see Baniqued et al., 2014). Each game was selected based on their correlation with WM and reasoning abilities derived from a single session of game play (Baniqued et al., 2013, 2014). Although the nature and magnitude of the game vs. WM and reasoning relationships did not differ between the two groups at baseline, the groups differed in game structure—a distinction that could become important when examining cognitive ability relationships across extended gameplay. In one group, players solved novel and increasingly challenging problems in order to progress to each new level. At each subsequent training session, players in this group started on the last level they had reached in the previous session (i.e., puzzle-based games played adaptively across sessions). In the second group, players quickly switched attention to different game components (e.g., falling coins or numbers) in order to reach the highest score possible, with increasing components or switching demands at each new level. However, unlike the first group, players started at the same level at each session and after each “game over” or failed attempt (i.e., speeded switching games played non-adaptively across sessions).

We used baseline cognitive assessments and game data from participants' first and final training sessions and to explore how game performance-cognitive ability relationships change with game structure and extended gameplay. We then examined the unique predictive ability of fluid abilities commonly used in skill acquisition by running a series of step-wise regression models with both working memory and reasoning as predictors. Thirdly, we explored the unique predictive ability of perceptual speed, given its importance in later stages of complex skill acquisition (e.g., Ackerman, 1988; see Ackerman and Cianciolo, 2000, Experiment 3 for similar regression analyses using first and final session complex task performance metrics).

## Materials and methods

In these analyses, we used a subset of participants from a cognitive training study that tested the effects of casual game training on cognitive performance (Baniqued et al., 2014). We used data from participants in the “WM-Reas 1” (non-adaptive) and “WM-Reas 2” (adaptive) training groups that played working memory and reasoning games. Brief descriptions of the procedure for this subset of participants are provided in the next section. For a detailed description of the study procedures, see Baniqued et al. (2014). All procedures were approved by the University of Illinois Institutional Review Board.

### Participants

Participants ranging from 18 to 30 years in age were recruited from online postings, flyers, and newspaper advertisements. Respondents were screened with several criteria including a prerequisite of 3 h or less of video and board game play per week in the last 6 months. All participants signed a consent form approved by the University of Illinois Institutional Review Board. Participants who completed the study were paid $15 an hour, and if they dropped out at any point during the study they were paid $7.50 an hour for the time that they had completed. Out of the 276 participants recruited for the casual games project, here we use a subset of data containing 128 individuals, corresponding to the two groups relevant to the current study.

After qualifying for the study, participants were randomized into one of the groups with one caveat: halfway through data collection, while participants were undergoing testing or training as part of the non-adaptive group or another active control group (latter not included in the current analysis), we included the adaptive game group and a no-contact control group (see Baniqued et al., 2014 for more details). After reaching ~50 participants with complete data in each of the non-adaptive group and the active control group, we stopped randomizing additional participants into these two groups but continued randomizing participants into either the adaptive group or no-contact control group (the latter not included in the current analyses but see Baniqued et al., 2014).

At the halfway point mentioned previously, we also included Symmetry Span to fully capture a more general working memory construct. Therefore, only the subjects in the non-adaptive group (and in the no-contact group that is not included in this analysis) completed the Symmetry Span task (see Section Working Memory and **Table 2**).

Table 1 shows the demographic information for participants included in these analyses.

**Table 1:** **Participant demographics**.

	**Non-adaptive**	**Adaptive**
*N* started but did not complete study	11	12
*N* excluded due to game play >3 h per week	3	1
*N* excluded due to casual games played outside training	2	5
*N* included in analyses	48	46
Males	15	15
Age	21.29 (2.20)	21.17 (2.51)
Years of education	14.93 (1.34)	14.89 (1.77)

*N, Number of participants. Standard deviation is noted in parentheses*.

### Procedure

After group assignment, participants underwent four testing sessions consisting of three cognitive sessions and one magnetic resonance imaging (MRI) testing session administered in a fixed order. The neuroimaging data is not discussed in this paper. After baseline testing, participants completed ten video game training sessions at a rate of two to three times per week. Each game was played for 20 min per session, with each session lasting around 1.5 h. At the end of training, re-testing was completed to assess transfer of cognitive task skills; transfer analyses are reported elsewhere and not the focus of the current study (Baniqued et al., 2014). The current analyses excluded participants based on video game play outside the laboratory using the same criteria as Baniqued et al. (2014) (Table 1).

The cognitive measures used as predictors in this study are from the tasks administered during the baseline testing sessions, while the casual game scores are derived from game performance in the first and final training sessions.

**Figure 1:**
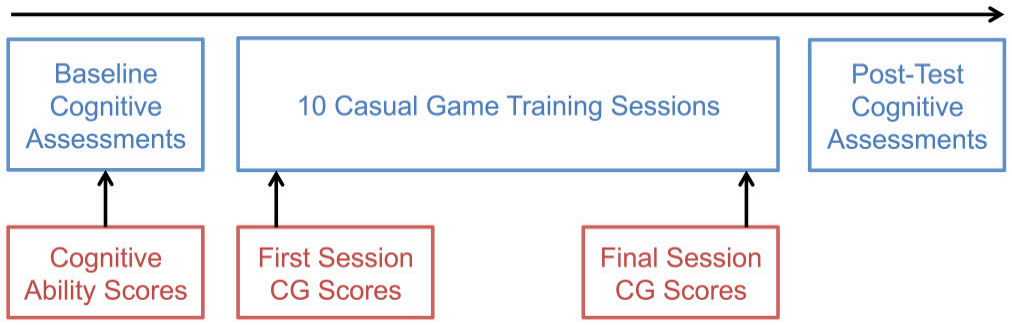
**General procedure for the entire casual game project (in blue) and the metrics for the current primary analysis (in red)**. Note, that the post-testing cognitive assessments were not used since the focus of the current study was on using *baseline* cognitive abilities to predict casual game performance across time.

Figure 1 summarizes the measures used in the longitudinal design of the casual game project.

### Baseline cognitive tasks

Tasks administered during baseline testing were divided into three categories: reasoning, working memory, and perceptual speed. Below are brief descriptions of each task, with more details in the Supplemental Material of the published training report (Baniqued et al., 2014).

#### Reasoning

All tasks except Matrix Reasoning were taken from the Virginia Cognitive Aging Project (VCAP; see Salthouse and Ferrer-Caja, 2003; Salthouse, 2004, 2005; Salthouse et al., 2008). Matrix reasoning was based on Ravens (1962) and Crone et al. (2009) and performed in an MRI environment. All tasks were completed on a computer with the exception of the Shipley Abstract test, which was administered with paper and pencil.

##### Form boards (Ekstrom et al., 1976)

Participants were instructed to select shapes to exactly fill the area of a bigger shape on a computer. The dependent measure was the total number of correctly completed problems within 8 min.

##### Letter sets (Ekstrom et al., 1976)

Participants viewed five patterns of letter strings and were instructed to choose the string that does not match the other four strings. The dependent measure was the total number of correctly completed problems within 10 min.

##### Paper folding (Ekstrom et al., 1976)

Participants attempted to identify the resulting pattern of holes from a sequence of folds and a punch through the folded sheet. The dependent measure was the total number of correctly completed problems within 10 min.

##### Spatial relations (Bennett et al., 1997)

Participants selected a two dimensional unfolded object that matched a three-dimensional folded object. The dependent measure was the total number of correctly completed problems within 10 min.

##### Shipley abstract (Zachary and Shipley, 1986)

Participants filled in missing item(s) to complete progressive sequences of numbers, letters, and words written on one sheet of paper. Participants were instructed to attempt to complete all 20 sequences in 5 min. Participants were allowed to skip and revisit problems. The dependent measure was the total number of correctly completed problems.

##### Matrix reasoning (Ravens, 1962; Crone et al., 2009)

Participants viewed a 3 × 3 matrix containing patterns in all but one cell and were instructed to choose an item that best completes the pattern. There were 30 control trials in which no integration was required and 30 reasoning trials in which successful completion required integrated patterns across the cells. Participants had 12 s to solve each problem. If a response was not made after 12 s, subjects were prompted to respond. The dependent measure was the mean accuracy of the reasoning trials.

#### Working memory

Although there is some consensus for reasoning and perceptual speed as general constructs (Ekstrom et al., 1976; Schaie et al., 1989; Fry and Hale, 2000; Conway et al., 2002; Salthouse and Ferrer-Caja, 2003; Salthouse, 2004, 2005; Salthouse et al., 2008; Redick et al., 2012b), the WM construct is less clear. In the current study, we used a combination of tasks defined in various studies as “working memory tasks” to create a measure of general WM ability (see Wilhelm et al., 2013; Schmiedek et al., 2014)^2^.

##### Visual short term memory (Luck and Vogel, 1997)

A probe array of four shapes briefly appeared on the screen. After a delay, a target shape appeared and participants had to decide whether this stimulus was in the probe array. The experiment consisted of three blocks with stimuli varying only in color on the first block, only in shape on the second block, and the conjunctions of both color and shape on the third block. Each block consisted of 60 trials. The dependent measure was overall accuracy.

##### Spatial working memory (Greenwood et al., 2005; Erickson et al., 2011)

Each trial consisted of a configuration of one, two, or three black dots on the screen. After a brief delay, a red target dot appeared, and participants were instructed to determine if the red dot was in the same position as one of the black probe dots in that trial. There were 40 trials (20 same and 20 different) per condition randomly varying in dot locations and condition. The dependent measure was overall accuracy.

##### N-back (Kirchner, 1958; Kane et al., 2007)

For three blocks of trials, participants viewed a sequence of centrally presented letters. For each letter, participants were instructed to determine if the current letter matched the previous letter (first block, 1-back), two letters back (second block, 2-back), or three letters back (third block, 3-back). The most demanding condition, the 3-back condition, was used as a metric of working memory performance on this task. There were five 20-letter sequences per condition for a total of 100 trials (25 target trials for all conditions and 10 lure trials for the 2 and 3 back) per condition. The dependent measure was the mean accuracy across the two and three back conditions.

##### Running span (Broadway and Engle, 2010)

For each trial, a sequence of letters was rapidly presented on the screen. After the list was presented, participants were told to recall the last 2, 3, or 4 items in the sequence on the screen. The dependent measure was the number of correctly recalled items across all of the trials.

##### Symmetry span (Unsworth et al., 2005; Redick et al., 2012a)

Participants viewed a sequence of red squares within a matrix and in between presentation of the red squares, judged whether two figures were symmetrical. At the end of these sequences, participants were instructed to recall in order the locations of the previously presented sequence. There were three trials of list lengths of 2, 3, 4, and 5, for a total of 12 trials. The dependent measure was the number of correctly recalled items across all of the trials.

#### Perceptual speed

All tasks were from VCAP and completed with paper and pencil.

##### Digit symbol coding (Wechsler, 1997)

Participants were presented with nine unique symbols, each corresponding to a specific digit (1–9). They were then presented with a list of digits and instructed to write the corresponding symbol for each digit, completing as many items as possible within 2 min. The total number of correctly written symbols was used as the dependent measure.

##### Pattern comparison (Salthouse and Babcock, 1991)

Participants were asked to determine whether a pair of patterns is the same or different. Participants completed two sets of patterns and for each set, were given 30 s to match as many pattern pairs as possible. The average of correctly answered items across the two sets was used as the dependent measure.

##### Letter comparison (Salthouse and Babcock, 1991)

Participants were asked to determine whether a pair of letter strings is the same or different. Participants completed two sets of letter strings and for each set, were given 30 s to match as many letter string pairs as possible. The average of correctly answered items across the two sets was used as the dependent measure.

#### Composite cognitive scores

For each individual, each baseline cognitive measure was first standardized (i.e., *z*-scored using the mean and standard deviation collapsed across the two groups). For each individual, task measures were then averaged together with other measures of the same cognitive construct (based on the model-based grouping listed above for Reasoning, Working Memory, Perceptual Speed). Although WM and reasoning were the main focus of analyses—given that casual games were selected based on their associations with WM and reasoning, the relationship with perceptual speed scores were also analyzed given the construct's previous implications in skill acquisition (e.g., Ackerman, 1988).

### Casual games used for training

For both groups, four casual games previously associated with WM and reasoning (Baniqued et al., 2013, 2014) were each played for 20 min in a pseudo-random order for each of the 10 training sessions. The original training study did not explicitly manipulate game type and adaptive-ness for the two groups; their groupings for the purposes of this study are defined post-hoc. For brevity, we refer to the puzzle-based games played adaptively across sessions as the *adaptive games* and the speeded switching games played non-adaptively across sessions as the *non-adaptive games*.

For the adaptive group, common to each game was the goal to complete as many levels or stages as possible within the 20 min session. Participants needed to complete one level before advancing to the next. At the end of the 20 min, the current level was recorded as the high level for that session and used as the performance metric. An experimenter recorded this level information, which was then entered at the beginning of the next session. One game, Aengie Quest, was excluded from analyses as the majority of participants completed all the levels before the end of the training sessions. After data collection, video recordings for each game in each session were reviewed to ensure that the correct procedure was followed. That is, each participant must start on the level they were attempting from the previous session. If a subject did not start on the correct level (e.g., started on the first level instead of a higher level from a previous session), the data for that game was not included in calculating either casual game (CG) score composite measure (see Section Casual Game First and Final Session Composite Scores for composite CG score explanation).

For the non-adaptive group, one game was adaptive across sessions and was thus left out of analyses (Silversphere). For each session in the remaining three games, participants started over at the first level of each game, or the game was structured such that within a session, participants completed several attempts with each attempt starting from the first level of difficulty. The score of each game attempt was collected from the video recordings. If no video recording was obtained for either the first or final training session of a game, that game was excluded for both first and final CG composite scores (see Section Casual Game First and Final Session Composite Scores for composite CG score explanation).

Two subjects from both the adaptive and the non-adaptive groups were excluded from analyses because two out of three games had excluded or missing data for first or final session scores.

Below are descriptions of the games used in the current study.

#### Adaptive games

##### Silversphere (miniclip.com)

Players must move a sphere across a platform to a blue vortex without falling off the platform and within a short time limit, which ranges from around 30 s to 2 min across levels. Various objects block the path or help create a path to the blue vortex. Across levels, difficulty increases with new, more challenging objects and/or a greater number of objects to consider.

##### Blockdrop (miniclip.com)

Players move around a gem on three-dimensional blocks to remove all blocks except a checkered block. Each higher level presents unique and more complex block arrangements.

##### Gude balls (bigfishgames.com)

To complete each level, players must fill all plates with four of the same colored balls by rapidly moving and switching balls to other plates. Rails connect these plates and many contain obstacles, hindering a direct path to another plate. The introduction of unique obstacles, a greater number of plates, and shorter time limits increase the game's difficulty.

For all three games, the performance metric was the highest level reached during the session.

#### Non-adaptive games

##### Digital switch (miniclip.com)

In the main game, participants must collect falling colored coins by lining up the colored digibot switches with the correct color. After a game is over, players start on level 1. At each level, the number of coins to be collected increases by five coins and more coins fall simultaneously, requiring players to switch more quickly and attend to more coins at once. Highest score achieved was the metric used.

##### Two three (armorgames.com)

Participants play as a tank and must shoot down rapidly presented numbers by using a mouse to point the tank at the numbers and subtract the presented numbers down to exactly 0 using units of 2 and 3. These subtractions are achieved by aiming at the falling numbers and typing in 2 or 3 on the keyboard. When a number is hit, the number is reduced by the amount specified (2 or 3). If a number is not correctly subtracted down to exactly 0, it hits the player's tank, and the tank moves up the game screen. When the tank reaches the top of the game screen, the game is over. Participants then restart the game at first level with 0 points. As the game proceeds, more numbers are presented and the magnitude of presented numbers increases. Highest score achieved in the session was the metric used.

##### Sushi go round (miniclip.com)

Participants must earn money and a good reputation by completing restaurant tasks in a timely fashion. These include learning to prepare different recipes correctly, serving items to customers within a reasonable time frame, cleaning tables to make way for new customers, and ordering ingredients to keep up with demand. If participants achieve a specified amount of money by the end of the allotted time while maintaining a certain reputation level, they keep this money and advance to the next level. If players do not achieve these goals, the game is restarted on the first level with no money. At each consecutive level, the amount of money and/or the level of reputation needed to earn and maintain increases. The highest amount of money collected was the metric used.

#### Casual game first and final session composite scores

Training session 1 metrics were standardized and averaged together to create a composite first session CG score. Training session 10 metrics were standardized and averaged together to create a composite final session CG score.

## Results

### Descriptive statistics

Descriptive statistics for each cognitive test measure and casual game measure are reported in Tables 2, 3, respectively. The cognitive measures used in these analyses are from the baseline testing sessions only, while the casual game measures are from the training sessions. The two groups did not significantly differ on any of the individual measures or composite scores, according to an independent samples *t*-test (all *p*s > 0.05). Participants reached significantly higher scores after training (final session scores) compared to first session scores (all *p*s < 0.001).

**Table 2:** **Baseline cognitive test measures**.

**Cognitive ability**	**Task**	**Measure**	**Adaptive *M* (*SD*)**	**Non-adaptive *M* (*SD*)**	**Baseline group differences**
Reasoning	Matrix reasoning	% accuracy	78.59 (9.28)	79.86 (8.48)	*t*_(88)_ = 0.73, *p* = 0.47
Reasoning	Form boards	Total correct	9.8 (3.93)	9.6 (4.35)	*t*_(88)_ = −0.42, *p* = 0.68
Reasoning	Paper folding	Total correct	8.84 (1.97)	8.19 (2.36)	*t*_(88)_ = −1.65, *p* = 0.10
Reasoning	Spatial relations	Total correct	12.36 (4.13)	11.77 (4.34)	*t*_(88)_ = −0.85, *p* = 0.39
Reasoning	Letter sets	Total correct	12.56 (1.63)	12.35 (1.78)	*t*_(88)_ = −0.72, *p* = 0.47
Reasoning	Shipley abstract	Total correct	15.33 (2.27)	15.81 (2.16)	*t*_(88)_ = 1.23, *p* = 0.22
WM	SPWM	% accuracy	0.87 (0.07)	0.88 (0.07)	*t*_(86)_ = 0.87, *p* = 0.39
WM	Nback	% accuracy	0.86 (0.09)	0.88 (0.06)	*t*_(86)_ = 1.06, *p* = 0.29
WM	VSTM	% accuracy	0.81 (0.06)	0.8 (0.06)	*t*_(88)_ = −0.80, *p* = 0.42
WM	Running span	Total correct	22.49 (5.57)	21.79 (5.36)	*t*_(87)_ = −0.22, *p* = 0.83
WM	Symmetry span^*^	Total correct	18.89 (6.71)	16.92 (8.76)	*t*_(65)_ = −0.8, *p* = 0.43
Perceptual Speed	Pattern comp	Total correct	20.74 (3.4)	21.52 (4)	*t*_(88)_ = 0.96, *p* = 0.34
Perceptual speed	Letter comp	Total correct	12.42 (2.32)	13.22 (2.48)	*t*_(88)_ = 1.49, *p* = 0.14
Perceptual speed	Digit symbol coding	Total correct	90.76 (13.28)	93.13 (14.07)	*t*_(88)_ = 0.89, *p* = 0.38
Composite	Reasoning	Std average	0.02 (0.65)	−0.02 (0.72)	*t*_(88)_ = −0.4, *p* = 0.69
Composite	Working memory	Std average	0 (0.53)	0 (0.59)	*t*_(88)_ = 0.07, *p* = 0.95
Composite	Perceptual speed	Std average	−0.12 (0.71)	0.11 (0.86)	*t*_(88)_ = 1.41, *p* = 0.16

M, Mean; SD, Standard Deviation;

* *Only 25 participants completed Symmetry Span for the Non-adaptive group since this measure was added halfway through data collection*.

**Table 3:** **Casual game first and final session scores**.

**Group**	**Games**	**First session *M* (*SD*)**	**Final session *M* (*SD*)**	**First and final session differences**
Adaptive	Silversphere	8.96 (2)	19.41 (3.24)	*t*_(43)_ = −30.188, *p* < 0.001
	Block drop	16.41 (3.38)	52.59 (8.42)	*t*_(42)_ = −31.131, *p* < 0.001
	Gude balls	4.46 (1.19)	14.76 (2.66)	*t*_(36)_ = −30.311, *p* < 0.001
Non-adaptive	Two three	535.38 (186.47)	1079.32 (225.23)	*t*_(43)_ = −13.268, *p* < 0.001
	Digital Switch	7078.3 (2279.85)	14410.21 (3782.55)	t(43) = −10.31, p < 0.001
	Sushi Go Round	2807.5 (1110.53)	6637.29 (794.25)	t(43) = −16.812, p < 0.001

*M, Mean; SD, Standard Deviation*.

#### Correlations between baseline cognitive scores and casual game scores

First we calculated bivariate correlations of all baseline composite measures and CG composite scores used in subsequent regression analyses (Table 4). In Table 4, the correlation values and significance indicators (uncorrected for multiple comparisons) as well as the bootstrapped confidence intervals are displayed in the upper portion of the matrix for the two game groups. A significant relationship between both fluid abilities, and both first and final session CG scores were observed in the two training groups. Importantly, there was no evidence that the relationships between first session CG scores and fluid abilities were different between groups (REAS: *Z* = 0.29, *p* = 0.77; WM: *Z* = 0.4, *p* = 0.69). Perceptual speed was significantly related to both first and final session scores for the non-adaptive group only. Consistent with previous studies (Kane et al., 2005; Ackerman et al., 2005), there was a strong relationship between working memory and reasoning. Furthermore, for both groups, final and first session CG scores were highly related.

**Table 4:** **Correlation matrices of composite scores for both groups**.

**Composite score**	**Reasoning**	**Working memory**	**Perceptual speed**	**First session CG score**	**Final session CG score**
**ADAPTIVE GAME GROUP**					
Reasoning		[0.38,0.76]	[−0.24,0.37]	[0.19,0.79]	[0.4,0.85]
Working memory	0.62^***^		[−0.17,0.49]	[0.02,0.65]	[0.4,0.78]
Perceptual speed	0.06	0.15		[−0.34,0.28]	[−0.21,0.38]
First session CG score	0.58^***^	0.42^**^	−0.05		[0.47,0.9]
Final session CG score	0.72^***^	0.62^***^	0.09	0.79^***^	
**NON-ADAPTIVE GAME GROUP**					
Reasoning		[0.21,0.68]	[−0.16,0.41]	[0.45,0.75]	[0.21,0.61]
Working memory	0.47^***^		[−0.03,0.61]	[0.24,0.67]	[0.12,0.61]
Perceptual speed	0.13	0.35^*^		[0.1,0.61]	[0.07,0.6]
First session CG score	0.62^***^	0.49^***^	0.39^**^		[0.45,0.8]
Final session CG score	0.43^**^	0.37^*^	0.37^*^	0.66^***^	

*** *p < 0.001*,

** *p < 0.01*,

* *p < 0.05, uncorrected for multiple comparisons. The upper triangle (for each group) contains the bootstrapped confidence intervals corresponding to the lower triangle of correlation values*.

**Figure 2:**
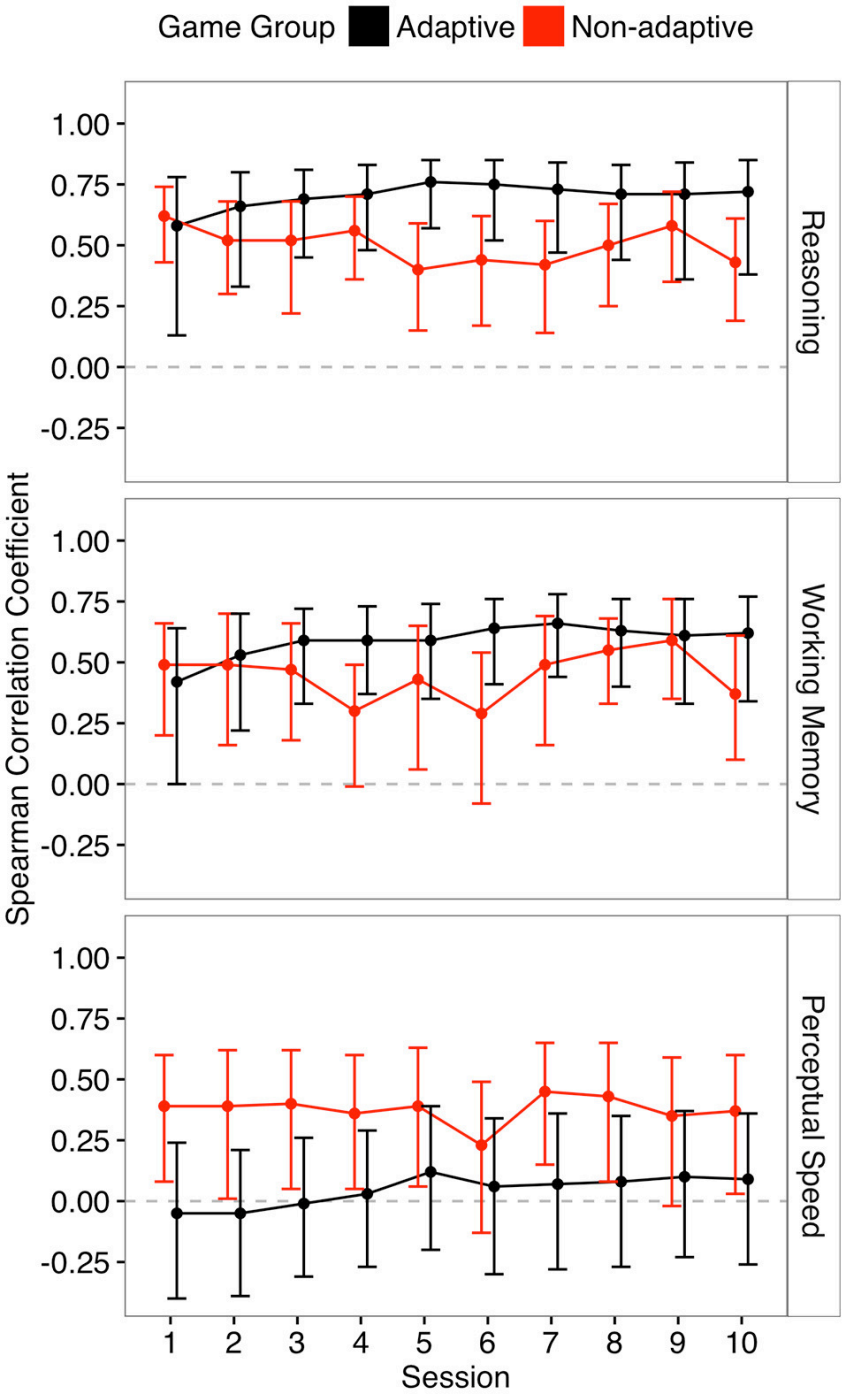
**Spearman correlation between game performance at each training session and each pre-training baseline measure**. Error bars are 95% bootstrapped confidence intervals.

Figure 2 contains the bivariate correlations for all training sessions including sessions not included in the main regression analyses (uncorrected for multiple comparisons). Supplemental Tables 1, 2 contain the bivariate correlations between each baseline measure (task and composite measures) and each individual game measure included.

#### Dynamics of cognitive ability casual game scores across game sessions

To assess how these relationships between cognitive abilities and casual game scores changed across time, and if this change differed between the two groups, we created three linear mixed effects models for each cognitive composite score. In each of these models, we included a random effect of subject. These models were implemented with the “lmerTest” package in R (Kuznetsova et al., 2015; R Core Team, 2015). Fixed effects parameters included main effects of cognitive score (reasoning, working memory, and perceptual speed), game session (final vs. first), and group (adaptive vs. non-adaptive) with all interaction terms included (cognitive score by session, cognitive score by group, group by session, and group by session by cognitive score). The three-way interaction of group, time and condition (Table 5) showed that both reasoning and working memory became more related to casual game scores from first to final session for the adaptive group compared to the non-adaptive group. Table 5 includes the fixed effects parameter estimates and model goodness of fit measures. These three-way interactions remained significant when including all 10 training sessions (Supplemental Table 3).

**Table 5:** **Model summaries predicting casual game scores**.

**Cognitive predictor**	**Reasoning**	**Working memory**	**Perceptual speed**
**FIXED EFFECTS**			
Cognitive	0.649^***^	0.613^***^	0.341^**^
	(−0.133)	(−0.171)	(−0.135)
Group	−0.058	−0.013	0.014
	(−0.136)	(−0.148)	(−0.167)
Session	−0.011	−0.005	−0.004
	(−0.085)	(−0.085)	(−0.087)
Cognitive^*^Group	0.098	0.021	−0.401^*^
	(−0.204)	(−0.26)	(−0.216)
Cognitive^*^Session	−0.199^*^	−0.157	−0.017
	(−0.118)	(−0.14)	(−0.101)
Group^*^Session	0.004	0.006	0.024
	(−0.122)	(−0.121)	(−0.125)
Cognitive^*^Group^*^Session	0.424^**^	0.503^**^	0.191
	(−0.182)	(−0.213)	(−0.163)
Intercept	0.019	−0.002	−0.039
	(−0.095)	(−0.104)	(−0.116)
**MODEL GOODNESS OF FIT MEASURES**			
Log Likelihood	−163.094	−171.424	−184.731
Akaike Information Criteria	346.188	362.847	389.462
Bayesian Information Criteria	378.117	394.777	421.392

* *p < 0.05*,

** *p < 0.01*,

*** *p < 0.001, uncorrected for multiple comparisons. The fixed effects values represent the parameter estimates of each model with the corresponding standard error of the mean in parentheses*.

#### Predicting first and final session casual game scores

To examine the extent to which working memory and reasoning predicted casual game performance, multiple sets of stepwise regression analyses were performed. Summary statistics for each added variable are reported in Table 6. All models and model comparisons were generated in R (R Core Team, 2015) with bootstrapped confidence intervals generated with the “boot” package (Canty and Ripley, 2015). The variance inflation factor for added variables in the final models were close to 1 and never above 10 (adaptive group: *M* = 1.53, Range = 1.26–1.84; non-adaptive group: *M* = 1.64, Range = 1.43–2.31), suggesting that multicollinearity was not a concern (Field et al., 2012). Added variable plots were created for each final model in each analysis to illustrate the unique effects of each predictor as well as to identify possible outliers and/or influential points (Supplemental Figure 1; Fox and Weisberg, 2011).

**Table 6:** **Summary of hierarchical regression analyses predicting casual game achievement using working memory and reasoning**.

**Variable added**	**Adaptive**			**Non-adaptive**		
	**β [*BCA* 95% *CI*]**	**Adj *R^2^***	***p*(Δ*F*)**	**β [*BCA* 95% *CI*]**	**Adj *R^2^***	***p(ΔF)***
**PREDICTING FIRST SESSION**						
Step 1: Reasoning	0.58 [0.18, 0.78]	0.32		0.62 [0.44, 0.73]	0.37	
Step 2: WM	0.1 [–0.23, 0.41]	0.31	0.53	0.26 [–0.03, 0.55]	0.41	0.05
**PREDICTING FIRST SESSION**						
Step 1: WM	0.42 [0.04, 0.64]	0.16		0.49 [0.26, 0.67]	0.23	
Step 2: Reasoning	0.52 [0.13, 0.79]	0.31	<0.001	0.5 [0.22, 0.71]	0.41	<0.001
**PREDICTING FINAL SESSION**						
Step 1: Reasoning	0.72 [0.41, 0.84]	0.51		0.43 [0.22, 0.62]	0.17	
Step 2: WM	0.29 [0.07, 0.53]	0.55	0.03	0.21 [–0.08, 0.53]	0.18	0.17
**PREDICTING FINAL SESSION**						
Step 1: WM	0.62 [0.42, 0.79]	0.38		0.37 [0.14, 0.6]	0.12	
Step 2: Reasoning	0.54 [0.24, 0.76]	0.55	<0.001	0.33 [–0.01, 0.54]	0.18	0.04
**PREDICTING FINAL SESSION**						
Step 1: First	0.79 [0.48, 0.9]	0.62		0.66 [0.47, 0.81]	0.43	
Step 2: Reasoning	0.4 [0.12, 0.72]	0.72	<0.001	0.03 [–0.22, 0.37]	0.41	0.83
Step 3: WM	0.23 [0.04, 0.46]	0.74	0.02	0.05 [–0.25, 0.3]	0.4	0.72
**PREDICTING FINAL SESSION**						
Step 1: First	0.79 [0.5, 0.9]	0.62		0.66 [0.45, 0.8]	0.43	
Step 2: WM	0.35 [0.14, 0.65]	0.71	<0.001	0.05 [–0.22, 0.34]	0.41	0.68
Step 3: Reasoning	0.26 [0.03, 0.6]	0.74	0.02	0.02 [–0.26, 0.32]	0.4	0.9

*WM, Working Memory; First, first session CG game scores. The p(ΔF) column lists the p-value for the change in the F-statistic between models, uncorrected for multiple comparisons*.

##### First session CG scores: WM and reasoning

**Figure 3:**
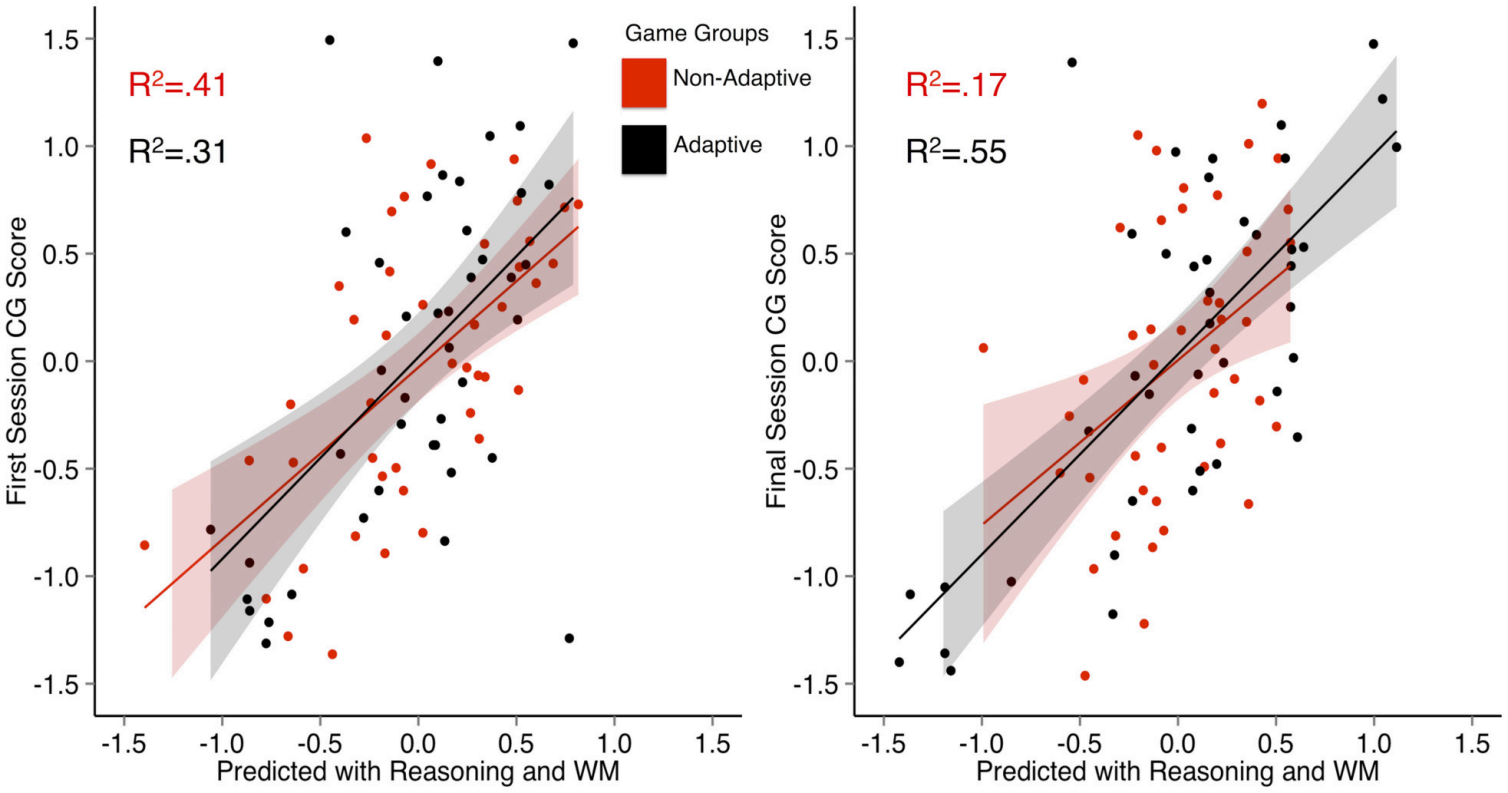
**Multiple regression plots showing the predicted values for first (left) and final (right) session casual game performance for non-adaptive and adaptive group games derived from regression models using WM and reasoning as predictors**. *R*^2^ = adjusted *R*^2^. The shaded area represents the 95% confidence region for each predictor in the model.

In a first set of stepwise regression analyses, first session CG scores were used as the outcome variable and cognitive scores were used as predictors. For the adaptive game group, reasoning emerged as the only significant predictor of first session CG scores (Table 6). For the non-adaptive game group, although some evidence for WM improving model fit existed in terms of a significant change in *F* (*p* = 0.05), this inference should be drawn cautiously as the bootstrapped confidence interval included 0. For the adaptive group full model, one possible outlier with a studentized residual value of −3.6 was identified. When excluding this subject from analyses, WM (β = 0.07, *p* > 0.05, *BCA* 95% *CI* [−0.24, 0.4]) remained insignificant and reasoning (β = 0.61, *p* < 0.001, *BCA* 95% *CI* [0.26,0.84]) remained significant. Figure 3 (left) shows each group's predicted first session CG scores from the full models (*p* < 0.001; including the previously investigated outliers).

##### Final session CG scores: WM and reasoning

In a second set of stepwise regression analyses, WM and reasoning were used as predictors of final session CG scores. Both WM and reasoning uniquely predicted final session CG scores (Table 6) in the adaptive group. Adding reasoning to a model with WM significantly improved the model fit, *F*_(1, 41)_ = 30.72, *p* < 0.001, while adding WM to a model with reasoning, *F*_(1, 40)_ = 4.45, *p* < 0.05 also improved model fit. One possible outlier with a studentized residual value of 4.0 was identified in the adaptive group full model. When excluding this subject from analyses, the WM (β = 0.27, *p* < 0.05, *BCA* 95% *CI* [0.03,0.53]) and reasoning (β = 0.63, *p* < 0.001, *BCA* 95% *CI* [0.35,0.82]) parameters remained significant. For the non-adaptive group, only reasoning significantly predicted final session scores (Table 6). Figure 3 (right) shows each group's predicted first session CG scores from the full models (*p* < 0.001; including the previously investigated outliers).

##### Final session CG scores: first session, WM, and reasoning

In another stepwise regression analysis, we examined the unique predictive ability of reasoning and WM over and above first session performance. First session CG scores were added to the models before the cognitive predictors. For the adaptive group, WM and reasoning uniquely predicted final session CG scores above and beyond first session CG scores (Table 6). We identified one potential influential point with a studentized residual of 3.2 and a cook's distance of 1.0. When excluding this subject from analyses, both WM (β = 0.16, *p* = 0.08, *BCA* 95% *CI* [−0.02,0.36]) and reasoning (β = 0.15, *p* = 0.15, *BCA* 95% *CI* [−0.05,0.36]) were marginally significant. This suggests some caution should be taken in interpreting these results. For the non-adaptive group, neither reasoning nor WM uniquely predicted final session CG scores above and beyond first session CG scores (Table 6).

Given that half of the non-adaptive group participants did not perform Symmetry Span, we performed the primary regression analyses (predicting casual game scores with reasoning and WM) with only those subjects who completed Symmetry Span and compared this to the analysis performed with subjects that were missing Symmetry span (Supplemental Table 4). In both of these groups, the findings and interpretations were similar for all of the above analyses.

##### First and final session casual game scores and perceptual speed

As a follow up analysis in the non-adaptive group, we added perceptual speed to the best fitting models using reasoning ability as the lone predictor, given that these non-adaptive games place greater demand on speed and accuracy of motor responses, and some evidence for a relationship was found in the previous correlation analysis (Table 4). Indeed, we found that perceptual speed predicted CG scores above and beyond reasoning ability for both first session CG scores (β = 0.31, *p* < 0.01, *BCA* 95% *CI* [0.09, 0.53]) and final session CG scores (β = 0.32, *p* < 0.05, *BCA* 95% *CI* [0.01, 0.55]). However, perceptual speed scores did not significantly predict final session scores over and above first session CG scores (β = 0.13, *p* > 0.05, *BCA* 95% *CI* [−0.09, 0.35]).

For comparison, we also performed these previous analyses with the adaptive group. Perceptual speed scores did not significantly predict adaptive CG scores in any model (*p*s > 0.05).

## Discussion

Cognitive training studies typically involve selecting games or tasks based on putative associations with specific cognitive abilities. Although informative, this approach overlooks potential changes in these relationships due to extended gameplay—changes that may have implications for the effectiveness of training targeted abilities. To shed light on this issue, we investigated the relationship between fluid abilities and casual game performance over time. In line with our previous study's findings (Baniqued et al., 2013), initial CG scores were robustly associated with WM and reasoning scores. The current analysis took a closer look at these relationships and found that reasoning and WM predicted relatively distinct aspects of performance over time. Specifically, (1) reasoning uniquely predicted first session CG scores for both the adaptive and non-adaptive games and accounted for the relationship with WM, (2) WM and reasoning uniquely predicted final CG scores for the adaptive game group, above and beyond first session CG scores, while (3) reasoning remained the only unique predictor of CG scores for the non-adaptive group.

Although WM and reasoning have been used to measure fluid abilities, they are rarely used together to understand the involvement of cognitive abilities in complex skill acquisition, despite recent evidence for their unique relationships with some complex tasks (Zook et al., 2004; Alloway and Alloway, 2010; Dumontheil and Klingberg, 2012). In the current study, using both WM and reasoning provided a richer understanding of the role of fluid abilities in CG performance over time. Specifically, reasoning, and much of the overlapping variance of WM, may be important for processes involved in novel task learning common to both the adaptive and non-adaptive games: finding solutions for novel game problems, integrating task instructions, and forming overall game strategies. In contrast, the unique predictive ability of WM was only evident in the adaptive games, when levels encountered later in training presumably placed greater demand on WM.

The current analyses show some support for the framework of complex skill acquisition, where task consistency is thought to moderate the relationship with cognitive abilities (Ackerman, 1988). For complex tasks with varied processing demands, the relationship to fluid abilities is thought to remain stable or increase over time as individuals remain in an effortful, cognitive stage of skill acquisition—a pattern exhibited by the adaptive game group that encountered novel rules and problems at each level. However, as stated previously, the emergence of WM as a unique predictor after several adaptive game sessions shows that different aspects of fluid abilities may become more important across tasks with varied processing demands. This distinction would not have been captured by use of a single measure of fluid ability or general cognitive ability (i.e., the common variance of all tasks).

Meanwhile, the relationships between cognitive abilities and CG scores in the non-adaptive group are more akin to tasks with consistent processing demands (also called consistent mapping; (Schneider and Shiffrin, 1977a,b)). Despite a decrease in both the overall sample variance for game performance and in the relationship to fluid ability over time, CG performance remained related to perceptual speed abilities over and above reasoning ability. In Ackerman's framework (Ackerman, 1988; Ackerman and Cianciolo, 2000), this is a pattern exhibited by consistent mapping tasks that emphasize speeded motor responses. Consistent processing demands likely emerge because the same strategies can be deployed over many instances (in this case, repeated levels), leading to automaticity (Logan, 1988). Thus, the stable association between perceptual speed and non-adaptive CG scores may reflect how quickly individuals deploy these learned strategies across training. For example, in the game Two Three, participants reported using a strategy of shooting down larger numbers as quickly as possible until the numbers were reduced to smaller, more manageable numbers (http://lbc.beckman.illinois.edu/pdfs/CasualGames_SuppAnalyses.pdf). In such cases where the same strategy is deployed at every level, higher difficulty levels (e.g., larger numbers to subtract on Two Three) may not involve a stable or increasing relationship with fluid abilities. This highlights an important distinction between task consistency and difficulty in adaptive tasks.

Other potential differences between individual casual games and groups deserve further consideration. Firstly, each group differed in adaptiveness *and* other cognitive processes common to each group's games (i.e., problem solving for the adaptive group and speeded, switching demands for the non-adaptive group). Therefore, the precise effect of adaptiveness or such common cognitive processes requires future work. Furthermore, although games in each group were classified by the aforementioned common game elements, some of the adaptive games had speed and attention switching demands similar to the non-adaptive games. For example, Gude Balls and Silversphere, but not Block Drop, involved demanding time constraints, speeded responses, and frequent shifts of attention to complete each level. Future studies may include identifying other game elements that affect the involvement of specific cognitive abilities. Moreover, examining different aspects of executive function (e.g., Miyake et al., 2000; Unsworth et al., 2014) or content specific processes (e.g., verbal and non-verbal WM specific storage and strategies compared to a general WM capacity; Kane et al., 2004) may identify critical unique and common cognitive components important for predicting game performance over time.

The current study also provides potential directions for cognitive training designs with casual games. Many cognitive training studies seek to train general fluid abilities or skills common to WM and reasoning (e.g., studies train on WM tasks to improve reasoning as in Jaeggi et al., 2008, 2011; Harrison et al., 2013). In the current study, reasoning mediated the relationship between WM and first session CG scores across both game groups, suggesting that a common fluid ability was most challenged early in gameplay. In this light, future cognitive training studies using casual games or similar tasks may consider introducing more games throughout training to maximally engage fluid abilities, where successful performance requires learning novel task environments. Casual games—both adaptive and non-adaptive— associated with common fluid abilities is a valuable resource for this endeavor, given the influx of new games introduced each year (http://www.casualgamesassociation.org) and the large collections of games already freely available.

In conclusion, the current study shows how the relationship of different types of casual games and cognitive abilities can change after prolonged gameplay. More generally, we show that the importance of certain cognitive abilities in video game performance may change differentially for different types of games, adding to a relatively limited literature on the relationship between cognitive abilities and video games (see Quiroga et al., 2009, 2011, 2015; Ackerman et al., 2010). Most importantly, this study illustrates how game elements and structure should be consideredwhen using complex games or tasks to improve or measure cognitive abilities.

## Author contributions

MK, PB, MV, HL, AK conceived and designed research. MK, PB, MV, HL contributed materials and/or programmed tasks for research. MK, PB performed the research. MK analyzed the data. MK, PB, AK wrote the paper.

## Funding

The Office of Naval Research supported this study (grant no. N000140710903).

### Conflict of interest statement

The authors declare that the research was conducted in the absence of any commercial or financial relationships that could be construed as a potential conflict of interest.

## Footnotes

^1^For the current study, we used absolute performance, rather than individual rate of change (e.g., learning curves or slopes). This metric was used to investigate how the relationship between casual game performance and cognitive abilities change rather than the relationship between a change (e.g., improvement) in casual game performance and cognitive abilities (for a discussion on this distinction, see Voelkle et al., 2006).

^2^We ran a principal component analysis (PCA) on the entire casual game project sample using the five task measures identified a priori as working memory. This PCA sample included the two groups of participants used for the primary analyses in this paper (adaptive, non-adaptive), as well as two additional groups (active control, no-contact control) that underwent the same battery of cognitive tests (excluding participants with >3 h of video game play; *N* = 258). Indeed, one principal component emerged with all variables contributing relatively equally to this common factor (Symmetry Span: 0.55, VSTM: 0.69 Spatial Working Memory: 0.68, Running Span: 0.49, N-Back: 0.63).
