# Supplementary material for: Examining the Roles of Reasoning and Working Memory in Predicting Casual Game Performance across Extended Gameplay
Source: Front Psychol. 2017 Mar 7;8:203. doi: 10.3389/fpsyg.2017.00203 (PMC5339312; doi:10.3389/fpsyg.2017.00203)
Supplement: Supplementary file 1 [file Presentation1.PDF]

Supplementary Material

Examining The Role of Reasoning and Working Memory in Predicting Casual Game Performance Across Extended Gameplay

Michael B. Kranz*, Pauline L. Baniqued, Michelle W. Voss, Hyunkyu Lee, Arthur F. Kramer

*** Correspondence:** Michael B. Kranz**,** Department of Psychology, Beckman Institute for Advanced Science and Technology, University of Illinois at Urbana Champaign, 405 North Mathews Avenue, Urbana, IL 61801, USA

email: mbkranz@illinois.edu

1. Supplementary Tables

Supplemental Table 1. Bivariate correlation coefficients of individual tasks and games for the adaptive group games

	Game Scores	Composite		Silversphere		Blockdrop		Gudeballs	
Cognitive Ability	Measure	First	Final	1	10	1	10	1	10
Reasoning	MatrixReasoning	0.27	.45**	.3*	.37*	0.24	.43**	0.17	0.17
Reasoning	FormBoards	.47**	.56***	.5***	.44**	0.29	.53***	0.27	.46**
Reasoning	PaperFolding	.39**	.42**	.39**	.4**	0.3	.37*	.35*	.35*
Reasoning	SpatialRelations	.53***	.63***	.63***	.6***	0.26	.63***	.36*	.37*
Reasoning	LetterSets	.54***	.47**	.33*	0.29	.48**	.49***	.49**	.35*
Reasoning	ShipleyAbstract	0.13	.33*	0.04	0.29	0.13	0.25	0.14	0.3
WM	SPWM	0.2	.38*	0.18	.42**	0.04	0.27	0.32	0.29
WM	Nback 3back	.43**	.52***	.5***	.38*	0.11	.43**	.5**	.53***
WM	VSTM	0.1	0.25	0.1	0.08	0.11	0.21	0.23	0.32
WM	Run Span	0.05	0.13	0.06	0.01	0.03	0.03	0.01	.44**
WM	Symmetry Span	.49***	.42**	0.29	0.3	.37*	.34*	.52***	.46**
Perceptual Speed	PatternComp	0.07	0.01	0.1	0.12	0.1	0.09	0	0.03
Perceptual Speed	LetterComp	0.04	0.07	0.21	0.03	0.02	0.06	0.17	0.21
Perceptual Speed	DSST	0.01	0.15	0.09	0.07	0.04	0.02	0.21	.42**
Composite	REAS	.58***	.72***	.54***	.6***	.42**	.68***	.37*	.51**
Composite	WM	.42**	.62***	.37*	.43**	0.12	.47**	.55***	.72***
Composite	PSpeed	0.05	0.09	0.18	0.07	0.05	0	0.16	0.26

Note. ***p<.001,**p<.01,*p<.05,First= first training session casual game performance, Final=final training session casual game performance, DSST=Digit Symbol Coding REAS=Reasoning, WM=Working Memory, PSpeed=Perceptual Speed.

Supplemental Table 2. Bivariate correlation coefficients of individual tasks and games for the non-adaptive group games

	Game Scores	Composite		TwoThree		DigiSwitch		Sushi	
Cognitive Ability	Measure	First	Final	1	10	1	10	1	10
Reasoning	MatrixReasoning	.43**	.31*	0.28	0.19	.46**	.43**	0.28	0.01
Reasoning	FormBoards	.57***	.38*	.32*	0.22	.62***	.4**	.38*	0.19
Reasoning	PaperFolding	.46**	.34*	.32*	0.24	.38*	0.27	.36*	0.26
Reasoning	SpatialRelations	.43**	.35*	0.13	0.19	.43**	.34*	.44**	0.19
Reasoning	LetterSets	0.23	0.2	0.15	0.12	0.27	0.15	0.14	0.14
Reasoning	ShipleyAbstract	.51***	0.24	0.25	0.09	.5***	0.27	.43**	0.21
WM	SPWM	0.21	0.21	0.07	0.15	.36*	0.24	0.08	0.03
WM	Nback 3back	0.27	.3*	0.11	0.17	0.23	0.2	0.29	.31*
WM	VSTM	0.28	.34*	.3*	0.15	0.2	0.26	0.18	.34*
WM	Run Span	0.17	0.08	0.1	0.18	0.11	0.15	0.2	0.13
WM	Symmetry Span^	.84***	0.22	0.4	0.24	.72***	0.12	.77***	0.07
Perceptual Speed	PatternComp	.53***	.43**	.44**	.31*	.39**	.48***	.39**	0.24
Perceptual Speed	LetterComp	0.02	0.1	0.06	0.11	0.11	0.1	0.11	0.09
Perceptual Speed	DSST	.4**	.37*	.35*	0.22	.47**	0.29	0.14	.37*
Composite	REAS	.62***	.43**	.34*	0.25	.62***	.44**	.48**	0.24
Composite	WM	.49***	.37*	.29*	0.26	.45**	.31*	.43**	0.24
Composite	PSpeed	.39**	.37*	.35*	0.26	.39**	.35*	0.17	0.28

Note. ***p<.001,**p<.01,*p<.05,First= first training session casual game performance, Final=final training session casual game performance, DSST=Digit Symbol Coding REAS=Reasoning, WM=Working Memory, PSpeed=Perceptual Speed. ^Only 24 participants completed Symmetry Span for the Non-Adaptive group as this measure was added half way through data collection.

Supplemental Table 3. Linear mixed models predicting standardized game performance across all 10 sessions.

Parameters	Reasoning	Working Memory	Perceptual Speed
Fixed Effects			
Cognitive	0.591***	0.567***	0.355***
	(-0.119)	(-0.155)	(-0.13)
Group	-0.065	-0.017	0.009
	(-0.123)	(-0.135)	(-0.161)
Session	-0.001	-0.0002	0.0002
	(-0.006)	(-0.006)	(-0.006)
Cognitive*Group	0.286	0.233	-0.418**
	(-0.183)	(-0.236)	(-0.209)
Cognitive*Session	-0.014*	-0.001	-0.003
	(-0.008)	(-0.009)	(-0.007)
Group*Session	0.001	0.001	0.003
	(-0.008)	(-0.008)	(-0.008)
Cognitive*Group*Session	0.029**	0.029**	0.025**
	(-0.012)	(-0.014)	(-0.011)
Intercept	0.014	-0.005	-0.043
	(-0.086)	(-0.094)	(-0.112)
Model Goodness of Fit Measures			
Log Likelihood	-487.175	-494.865	-510.042
Akaike Inf. Crit.	994.35	1009.73	1040.084
Bayesian Inf. Crit.	1042.352	1057.731	1088.085

Note. *$p < .05$, **$p < .01$, ***$p < .001$

2. Supplementary Figures

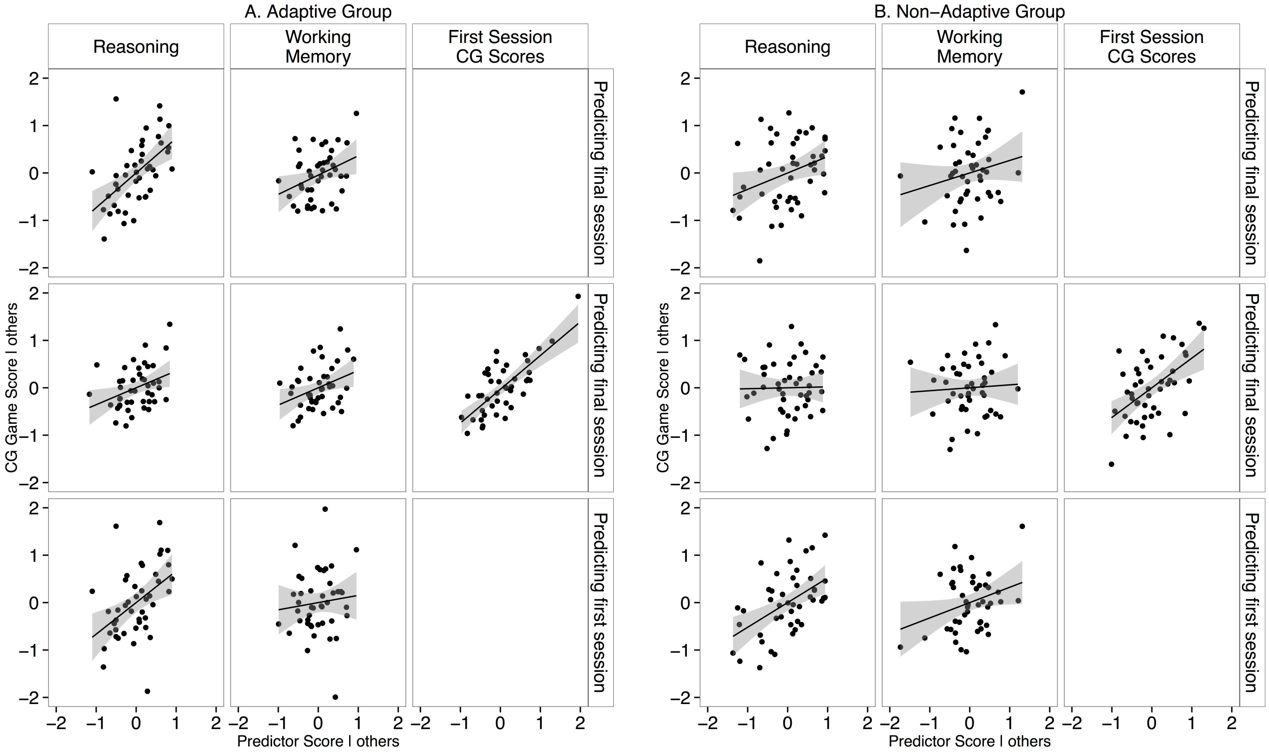

Supplemental Figure 1. Added variable plots for adaptive (A) and non-adaptive (B) game group full regression models. Along the x-axis is predictor score and the y-axis is standardized, composite game score after adjusting for the effects of the other predictors. Each panel column represents a predictor in each of these full models. Each panel row represents the dependent casual game variable for that model (top row: predicting first session game performance with reasoning and WM, middle row: predicting final session game performance with reasoning, WM and first session CG score predictors, bottom row: predicting final session game performance with first session game performance, reasoning, and WM predictors) The shaded area represents the 95% confidence region for each predictor in the model.
